# Reducing Virus Transmission from Heating, Ventilation, and Air Conditioning Systems of Urban Subways

**DOI:** 10.3390/toxics10120796

**Published:** 2022-12-17

**Authors:** Ata Nazari, Jiarong Hong, Farzad Taghizadeh-Hesary, Farhad Taghizadeh-Hesary

**Affiliations:** 1 Department of Mechanical Engineering, University of Tabriz, Tabriz 51666-16471, Iran; 2 Mechanical Engineering & Saint Anthony Falls Laboratory, University of Minnesota, Minneapolis, MN 55455, USA; 3 ENT and Head and Neck Research Center and Department, The Five Sense Health Institute, School of Medicine, Iran University of Medical Sciences, Tehran 14535, Iran; 4 TOKAI Research Institute for Environment and Sustainability (TRIES), Tokai University, Hiratsuka-shi 259-1292, Kanagawa-ken, Japan; 5 School of Global Studies, Tokai University, Hiratsuka-shi 259-1292, Kanagawa-ken, Japan

**Keywords:** indoor air quality, air circulation, aerosol, *OpenFOAM*, SARS-CoV-2, urban subway, ventilation

## Abstract

Aerosols carrying the virus inside enclosed spaces is an important mode of transmission for severe acute respiratory syndrome coronavirus 2 (SARS-CoV-2), as supported by growing evidence. Urban subways are one of the most frequented enclosed spaces. The subway is a utilitarian and low-cost transit system in modern society. However, studies are yet to demonstrate patterns of viral transmission in subway heating, ventilation, and air conditioning (HVAC) systems. To fill this gap, we performed a computational investigation of the airflow (and associated aerosol transmission) in an urban subway cabin equipped with an HVAC system. We employed a transport equation for aerosol concentration, which was added to the basic buoyant solver to resolve the aerosol transmission inside the subway cabin. This was achieved by considering the thermal, turbulent, and induced ventilation flow effects. Using the probability of encountering aerosols on sampling surfaces crossing the passenger breathing zones, we detected the highest infection risk zones inside the urban subway under different settings. We proposed a novel HVAC system that can impede aerosol spread, both vertically and horizontally, inside the cabin. In the conventional model, the maximum probability of encountering aerosols from the breathing of infected individuals near the fresh-air ducts was equal to 51.2%. This decreased to 3.5% in the proposed HVAC model. Overall, using the proposed HVAC system for urban subways led to a decrease in the mean value of the probability of encountering the aerosol by approximately 84% compared with that of the conventional system.

## 1. Introduction

Since December 2019, the novel coronavirus disease (COVID-19) has emerged as a significant global concern. It has led to 6,299,323 deaths worldwide as of 6 June 2022 and has evoked an urgent response from all disciplines to control the crisis [1,2,3,4,5]. Airborne transmission through droplet nuclei (aerosols) is an important mode of transmission of SARS-CoV-2 [6]. Airborne transmission depends on the distance traveled by the aerosol, congregation density of people, and ambient conditions, such as wind speed, temperature, relative humidity, particle size, and particle shape. Respiratory droplets and aerosols can be exhaled during coughing, sneezing, talking, or breathing [7,8,9,10,11,12,13,14,15,16,17,18,19,20,21,22,23,24,25,26,27]. To date, studies have evaluated SARS-CoV-2 spread through heating, ventilation, and air conditioning (HVAC) systems in various practical settings, such as underground car parks [28], restaurants [29], restrooms [30,31,32], elevators [33], public spaces [34], cleanrooms [35], urban buses [36], classrooms [37,38,39,40,41], cafeterias [42], airplanes [43], dental clinics [44,45], escalators [46], orchestral wind instrument performances [47], and other confined spaces [48,49]. Many studies have been conducted to investigate airborne transmission and develop appropriate mitigation strategies [50,51,52]. However, investigation of airborne transmission in subways is lacking. Subways are potential hotspots for airborne transmission, considering the high passenger density. Contact tracing has revealed cases where the infection transmission likely occurred during the subway transition [53].

Subway HVAC systems have been utilized to ventilate cabins for thermal comfort [53,54,55,56]. The conventional ventilation system of an urban subway comprises supply and exhaust ducts spanning the cabin ceiling and a mechanism for moving pollutants, such as breath aerosols toward the exhaust and recirculated-air ducts. Air is recycled through high-efficiency particulate air (HEPA) filters at the recirculation ducts. Air is constantly sucked up through vents, cooled, and filtered before being pushed back through supply air ducts [55]. Chang et al. demonstrated that such a subway HVAC system exhibits poor thermal comfort and energy-utilization efficiency and proposed a novel hybrid ventilation model to solve these problems [54]. Subway HVAC systems based on conventional exhaust ducts can spread respiratory droplets in the airflow direction and increase the risk of viral transmission. The number of passengers in the subway car and the exhaust duct configuration affect the risk of viral transmission. Given the characteristics of respiratory aerosol transmission on the HVAC streamline pattern, the virus can spread within an urban subway when a COVID-19 patient releases respiratory aerosols near the supply duct [53,55]. Urban subway ventilation redesign and passenger behavior modifications have rarely been studied directly in this setting.

Nazari et al. proposed antiviral adaptations for ventilation systems using jet fans inside underground car parks, focusing on the spread of respiratory droplets and aerosols and recommended several learning points to reduce the risk of viral transmission in this setting [28]. Liu et al. evaluated the effects of indoor airflow and associated aerosol transport on the risk of airborne viral transmission in a restaurant setting [29]. They identified that aerosols returning from HVAC systems, owing to low-efficiency filtration, can expose individuals near the outlet ducts to the possibility of infection. Dbouk and Drikakis [33] studied the impact of air-ventilation systems on airborne viral transmission in elevators and confined spaces. They concluded that the position of the inlets and outlets significantly influences the flow circulation and aerosol dispersion inside an elevator and indicated that using a single pair of inlet and outlet reduces droplet dispersion. Zhang et al. investigated the effects of wearing a face mask, opening windows and doors, and using an HVAC system on the aerosol spread inside an urban bus, and found that by opening doors and windows, the concentration of aerosols was reduced by approximately 50% [36]. Foster and Kinzel [41] estimated the risk of SARS-CoV-2 transmission in a classroom setting using computational fluid dynamics (CFD) and the Wells–Riley model. They showed that a well-designed HVAC system, face mask usage, reduction in the exposure time, and number of occupants are more important parameters than physical distancing. Wu et al. numerically studied virus dispersion from sneezing inside a cafeteria and stated that maintaining a safe distance in small indoor spaces (such as cafeterias) does not offer sufficient protection for activities without wearing face masks [42].

This study aimed to (i) provide a deep understanding of the routes of viral transmission in an urban subway setting, (ii) delineate the flaws of conventional subway HVAC systems and measures that enhance the transmissibility of virus-containing aerosols inside the cabin, and (iii) present mitigation strategies, including the design of a novel HVAC system to reduce the risk of viral transmission inside urban subways. We assumed the complete efficiency of HEPA filters inside the recirculation ducts in eliminating airborne viruses and focus on the airflow pattern inside the cabin and exhaust duct configuration. In the current study, we performed the simulations at a constant supply velocity within the range of typical values used in practical settings with various configurations of the breathing source.

## 2. Numerical Method and Simulation Setup

### 2.1. Formulation of the Subway HVAC Air Flows in the Cabin

The ventilation process of the urban subway HVAC system was modeled assuming continuous and incompressible air flow. To track the viral dynamics, the concentration equation was added to the basic solver to model the interactions between the fluid flow of the HVAC system and created aerosol concentration. In other words, the movement of small respiratory particles (<5 μm) originate from the continuous deformation of the cloud of respiratory particles with a shear flow interface induced by the HVAC system air. By solving the concentration transport equation, we achieved appropriate results for small respiratory particles (neglecting coughing and sneezing), considering gravity, drag, and temperature-dependent thermophoresis. Recently, Zhang et al. [36] and Nazari et al. [28] applied a concentration equation to simulate particle movement during human breathing. The relevant equations of motion are the continuity equation, momentum conservation law, and temperature equation, in their incompressible forms [28,36,39] as expressed in the following equations:

(1) $$div({\bar{u}}_{i})=0,$$

(2) $$\rho \frac{\partial ({\bar{u}}_{i})}{\partial t}+\rho \frac{\partial ({\bar{u}}_{i}{\bar{u}}_{j})}{\partial x_{j}}=-\frac{\partial \bar{p}}{\partial x_{i}}+g\cdot x\nabla (\frac{\rho }{\rho_{0}})+\frac{\partial }{\partial x_{j}}[\mu (\frac{\partial {\bar{u}}_{i}}{\partial x_{j}}+\frac{\partial {\bar{u}}_{j}}{\partial x_{i}})]+\frac{\partial }{\partial x_{j}}(-\rho {\bar{u}}_{i}^{\prime }{\bar{u}}_{j}^{\prime }),$$

(3) $$\rho \frac{\partial (\bar{T})}{\partial t}+\rho c_{p}(\frac{\partial }{\partial x_{j}}{\bar{u}}_{i}\bar{T})=\nabla (k\nabla \bar{T})+\frac{1}{2}\tau :(\nabla {\bar{u}}_{i}+\nabla {\bar{u}}_{j}^{T})+\frac{\partial }{\partial x_{j}}(-\rho {\bar{u}}^{\prime }{}_{i}\bar{T}),$$

(4) $$\rho \frac{\partial (\bar{C})}{\partial t}+\rho \frac{\partial ({\bar{u}}_{i}\bar{C})}{\partial x_{j}}=\nabla (D_{eff}\nabla \bar{C})+\frac{\partial }{\partial x_{j}}(\rho {\bar{u}}^{\prime }{}_{i}\bar{C}),$$

where *ρ*, *ρ_0_*, *p*, *u*, *C*, *T*, *k*, and $D_{eff}$ are the density, reference density, dynamic pressure, velocity, aerosol concentration, temperature, thermal conductivity, and diffusivity of the fluid, respectively. In Equation (3), the *Boussinesq* approximation is used for the momentum equation. In addition, in Equation (4), $D_{eff}$ = $\frac{\nu_{t}}{Sc_{t}}+\frac{\nu }{Sc}$, where *Sc_t_* and *Sc* are the turbulent and laminar Schmidt numbers, respectively, and *Sc_t_* = *Sc* = 1 [36]. In this study, a filter-based approach is used, which combines parts from both large eddy simulation (LES) and Reynolds-averaged Navier–Stokes equations (RANS) and compares the length of the characteristic with the mesh size-spatial filter to re-establish the turbulent viscosity as follows:

(5) $$\mu_{t}=FC_{\mu }\rho \frac{k^{2}}{\varepsilon },$$

where *k* and *ε* are the turbulent kinetic energy and the turbulent kinetic dissipation rate, respectively, and are derived directly from the standard *k–ε* two-equation turbulence closure model. Variable F is the filter function, which is defined in terms of filter size Δ as

(6) $$F=\min(1,C_{3\varepsilon }\frac{\Delta \varepsilon }{k^{\frac{3}{2}}}).$$

Substituting Equation (6) into Equation (5), we have

(7) $$\mu_{t}=C_{\mu }\min{(}_{}1,C_{3\varepsilon }\frac{\Delta \varepsilon }{k^{\frac{3}{2}}})\rho \frac{k^{2}}{\varepsilon }.$$

The filter used in Equation (6) modifies the standard *k-ε* model for a coarse filter size. In the far-field zone, the filter produces a hybrid RANS- LES behavior by allowing the development of length scales comparable to the grid resolution, and the turbulent viscosity is given as follows:

(8) $$\mu_{t}=C_{\mu }C_{3\varepsilon }\Delta \rho \sqrt{k},$$

where *C_3ε_* = 1 in the present case. To ensure that the numerically resolvable scale is compatible with the filtering process, the lower bound of the filter is set as $\Delta_{grid}$, where $\Delta_{grid}={\left(\Delta x\Delta y\Delta z\right)}^{\frac{1}{3}}$. The standard *k–ε* turbulence model is used in the following equations:

(9) $$\rho \frac{\partial (k)}{\partial t}+\rho \frac{\partial (k{\bar{u}}_{i})}{\partial x_{i}}=\frac{\partial }{\partial x_{j}}[\frac{\mu_{t}}{\sigma_{k}}\frac{\partial k}{\partial x_{j}}]+2\mu_{t}E_{ij}E_{ij}-\rho \varepsilon ,$$

(10) $$\rho \frac{\partial (\varepsilon )}{\partial t}+\rho \frac{\partial (\varepsilon {\bar{u}}_{i})}{\partial x_{i}}=\frac{\partial }{\partial x_{j}}[\frac{\mu_{t}}{\sigma_{\varepsilon }}\frac{\partial \varepsilon }{\partial x_{j}}]+C_{1\varepsilon }\frac{\varepsilon }{k}2\mu_{t}E_{ij}E_{ij}-C_{2\varepsilon }\rho \frac{\varepsilon^{2}}{k},$$

where $C_{\mu }=0.09$, $\sigma_{k}=1.00$,$\sigma_{\varepsilon }=1.30$, $C_{1\varepsilon }=1.44$ and $C_{2\varepsilon }=1.92$. The RSM turbulence model was used, as shown in equation (11). The following formula represents the partial differential equation (transport equation) for the stress tensor, which is derived from the Navier–Stokes equation as

(11) $$\begin{array}{l} \rho \frac{\partial ({\bar{u}}_{i}^{\prime }{\bar{u}}_{j}^{\prime })}{\partial t}+\rho \underset{C_{ij}}{\underset{︸}{\frac{\partial (u_{k}{\bar{u}}_{i}^{\prime }{\bar{u}}_{j}^{\prime })}{\partial x_{k}}}}=\underset{D_{T,ij}}{\underset{︸}{-\frac{\partial }{\partial x_{k}}[\rho {\bar{u}}_{k}^{\prime }{\bar{u}}_{i}^{\prime }{\bar{u}}_{j}^{\prime }+p(\delta_{kj}{\bar{u}}_{i}^{\prime }+\delta_{ik}{\bar{u}}_{j}^{\prime })]}}\\ +\underset{D_{L,ij}}{\underset{︸}{\frac{\partial }{\partial x_{k}}[\mu \frac{\partial ({\bar{u}}_{i}^{\prime }{\bar{u}}_{j}^{\prime })}{\partial x_{k}}]}}-\underset{P_{ij}}{\underset{︸}{\rho ({\bar{u}}_{k}{\bar{u}}_{k}^{\prime }\frac{\partial u_{j}}{\partial x_{k}}+{\bar{u}}_{j}^{\prime }{\bar{u}}_{k}^{\prime }\frac{\partial u_{i}}{\partial x_{k}})}}\\ -\underset{G_{ij}}{\underset{︸}{\rho \beta (g_{i}{\bar{u}}_{j}^{\prime }\bar{\theta }+g_{j}{\bar{u}}_{i}^{\prime }\bar{\theta })}}+\underset{\phi_{ij}}{\underset{︸}{p(\frac{\partial {\bar{u}}_{i}^{\prime }}{\partial x_{j}}+\frac{\partial {\bar{u}}_{j}^{\prime }}{\partial x_{i}})}}-\underset{\varepsilon_{ij}}{\underset{︸}{2\mu \frac{\partial {\bar{u}}_{i}^{\prime }}{\partial x_{k}}\frac{\partial {\bar{u}}_{j}^{\prime }}{\partial x_{k}}}}\\ -\underset{F_{ij}}{\underset{︸}{2\rho \Omega_{k}({\bar{u}}_{j}^{\prime }{\bar{u}}_{m}^{\prime }e_{ikm}+{\bar{u}}_{i}^{\prime }{\bar{u}}_{m}^{\prime }e_{jkm})}} \end{array}$$

where *C_ij_* is the convection term; *D_T,ij_* is the turbulent diffusion term; *D_L,ij_* is the molecular diffusion term; *P_ij_* is the stress production term; *G_ij_* is the buoyancy production term; φ_ij_ is the pressure strain term; *ε_ij_* is the dissipation term; and *F_ij_* is the production by the system rotation.

The particles potentially carrying SARS-CoV-2 have a size of 1–4 μm [57,58]. Aerosols within this size range have sufficient viral loads, and the effect of Brownian motion on aerosol movement is negligible [58]. Li et al. showed that Brownian force decreases rapidly with increasing particle diameter [59]. Moreover, they showed that the Brownian force becomes important only when the particle diameter is less than 0.3 μm. This feature can promote irregular movement of particles in space and enhance their diffusion ability. Some researchers have neglected Brownian motion to predict particle concentration distributions in rooms and obtained satisfactory results under flow settings similar to our current configuration [36,43]. To ignore the Brownian motion effect on aerosol diffusion, we assumed a Schmidt number equal to 1. This means that aerosols diffuse at the same rate as the momentum for 1–10 μm particles in the breathing model. This assumption is also used in the design of HVAC systems for clean rooms.

In the present study, we ignored the effects of spike-like structures (located at the viral surface) on viral transmission inside the urban subway because they are too small to affect the flow. Spike-like structures on the surface of SARS-CoV-2 may affect the dynamics of respiratory droplets if they are large [60]. Kanso et al. studied the effects of spike-like structures present on the surface of SARS-CoV-2 and their rotational diffusivity to attack a target cell. This study showed that the triangularity of the coronavirus spike bulb decreased its rotational diffusivity by 39%. They stated that SARS-CoV-2 particles rely on collisions with their surroundings to move into target cells. Kanso and co-workers studied a range of possible shapes of SARS-CoV-2 based on the virus size range and considered the rotational diffusivity and collision between them [60,61]. The applied concentration method for aerosol transmission inside an urban subway can encompass virus dispersion inside a confined space.

### 2.2. Numerical Technique and Boundary Conditions

In this study, we used the open-source field operation and manipulation (*OpenFOAM*) for computational fluid dynamics (CFD) software package version 5 to perform the numerical simulations. The *OpenFOAM* code is written in the C++ programming language and uses a finite-volume numerical technique to solve the conservation of mass, energy, momentum, and concentration equations, along with the equation of state, in their Reynolds-averaged forms.

**Figure 1:**
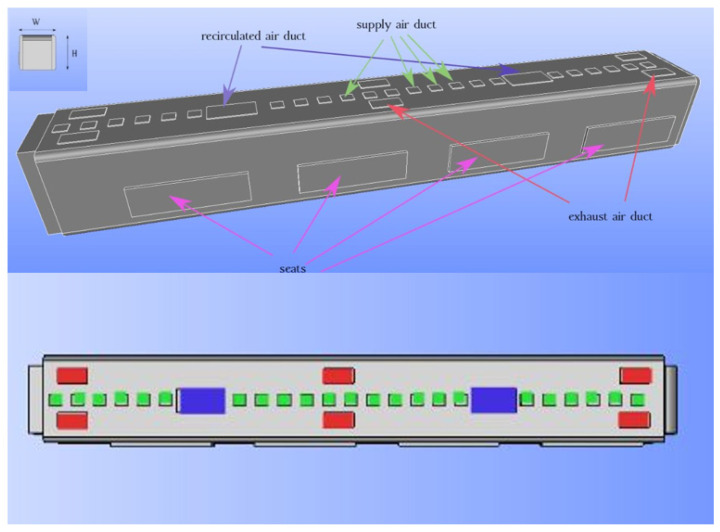
Schematic of a conventional urban subway car having supply air ducts, exhaust ducts, recirculated air ducts, and seats.

Numerical simulations were performed for the configuration of the ducts of an urban subway car, as shown in Figure 1. We applied a three-dimensional Cartesian coordinate system to govern the equations. The y-direction was set parallel to the length of the subway car; the z-direction was set opposite the direction of gravity; and the x-direction was set perpendicular to the subway car length (parallel to the width). The origin was located at the left corner of the subway car. The length, width, and height of the subway car were 21.14 m, 2.95 m, and 2.87 m, respectively. The heights of the standing and sitting mannequins in the simulations were set as 2 m and 1.4 m, respectively.

A Gaussian upwind scheme was used to handle the convective terms. A Gaussian linear-limited approach was employed to address the diffusion terms. The PIMPLE algorithm was applied to couple pressure and velocity. In addition, the maximum residuals for the convergence of pressure, velocity, temperature, and concentration were 10^−5^, 10^−8^, 10^−7^, and 10^−8^, respectively. To simulate aerosol movement within the urban subway, we ignored (i) the generation and (ii) interaction (collision) of particles due to the cloudy behavior of breath aerosols. The aerosol concentration is sensitive to the HVAC-induced airflow pattern, temperature, and ambient pressure. Among these environmental factors, relative humidity is a determinant parameter that strongly influences respiratory particle size during breathing. Various studies, such as those by Zhang et al. [36] and Liu et al. [29], demonstrated the traveling distance of respiratory particles based on their weight and size. The smallest particles were neutrally buoyant at a relative humidity of 20% and moved passively with the carrier fluid. Therefore, we ignored the Brownian motion of the particles and used a buoyant solver in *OpenFOAM*. In this method, gravity effects are considered instead of using the Lagrangian particle transport equation with Brownian motion. Zhang et al. [36] applied the buoyancy method to demonstrate respiratory particle transmission. However, both approaches are suitable.

**Figure 2:**
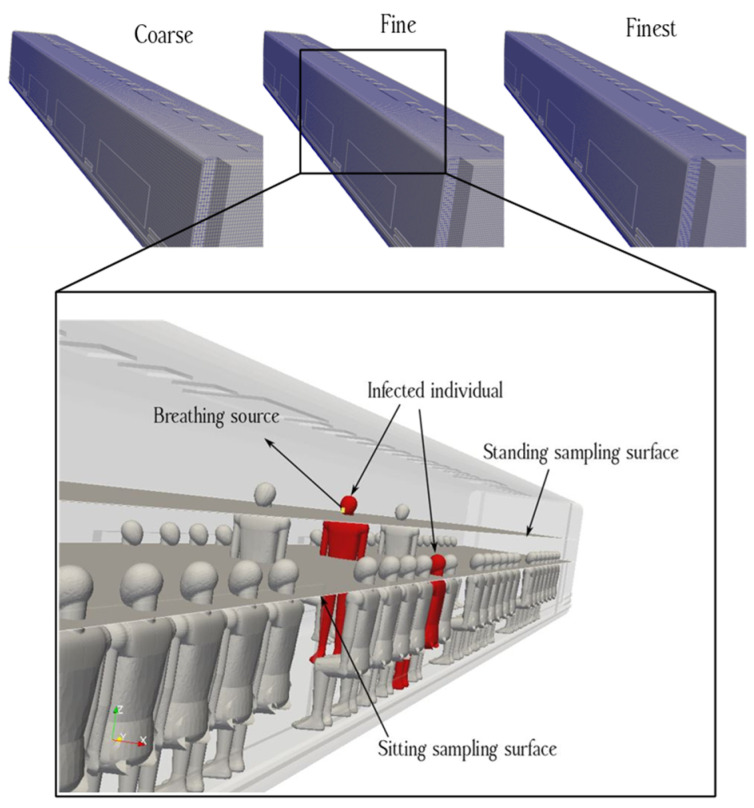
Schematic of a breathing source within the urban subway cabin, showing two mannequins (one standing and one seated) representing infected passengers.

**Figure 3:**
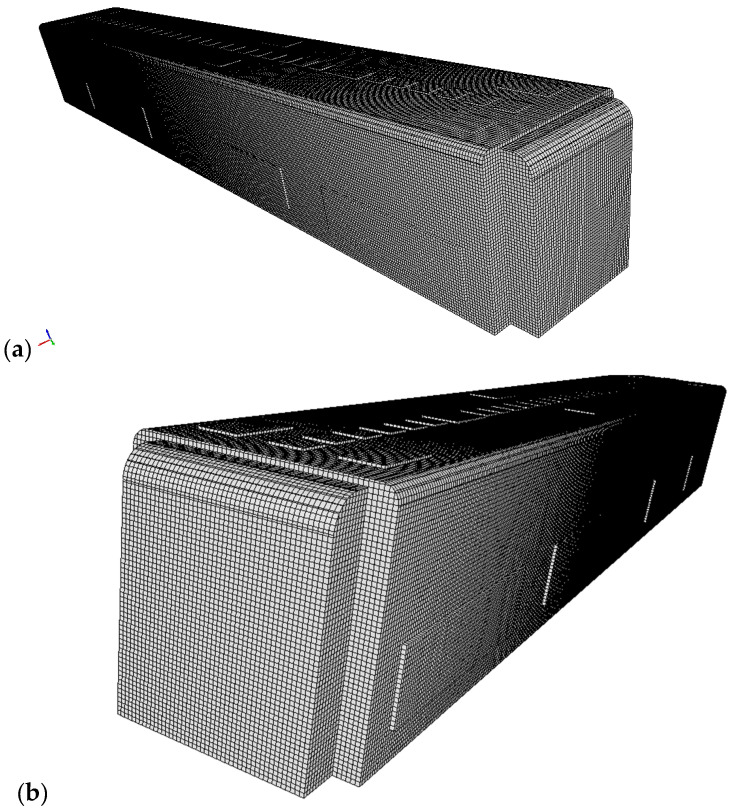

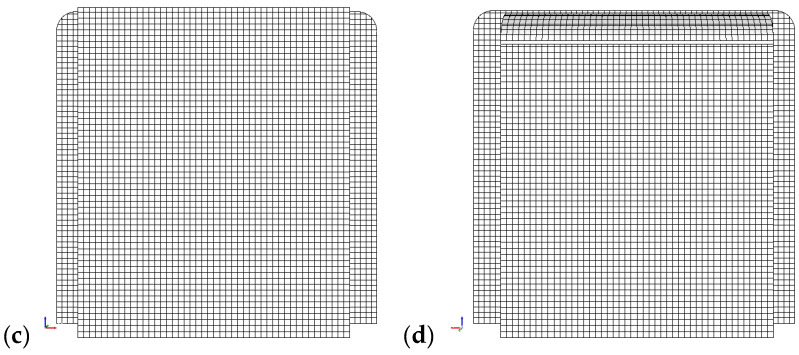
Schematic of mesh configuration for the present work. (**a**,**b**) are side views of urban subway fine mesh, (**c**) is the back view of fine mesh, and (**d**) is the front view of fine mesh.

**Figure 4:**
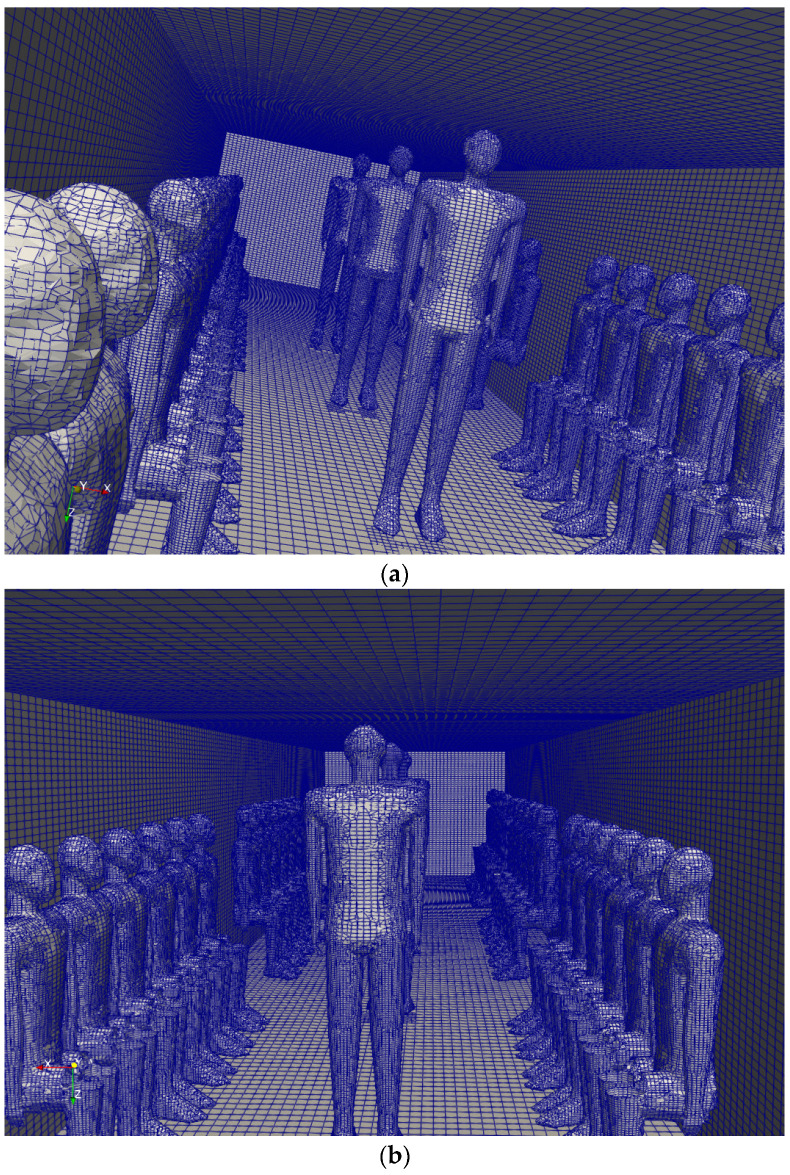
Schematic of mesh configuration for the present work, showing two mannequins. (**a**) the front view of the meshed mannequins, (**b**) the back view of meshed mannequins.

**Figure 5:**
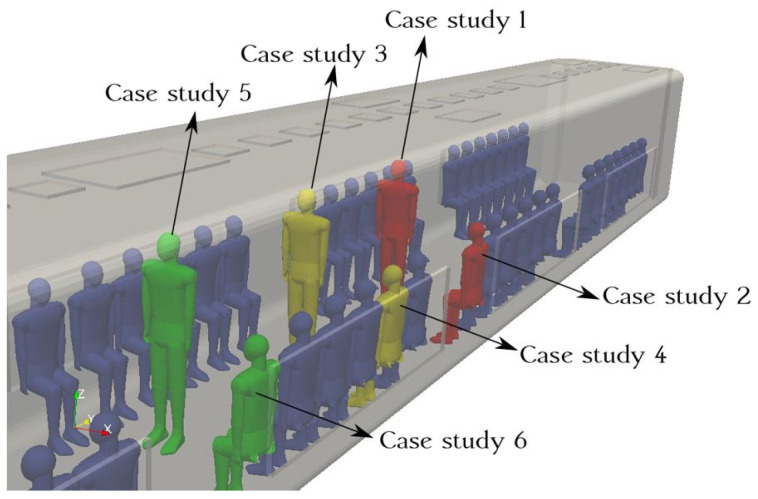
Positions and locations of the evaluated cases.

Figure 1 represents the three-dimensional (3D) duct configuration of an urban subway. Figure 2 shows the standing and sitting mannequins within the urban subway cabin having continuous breathing. In this study, we determined the standing and sitting sampling surfaces to calculate aerosol encounter probability (Figure 2). The sampling surfaces cross the centers of the passenger’s breathing zone hemispheres. The hemispherical breathing zone, having a radius of 300 mm, was defined as the area surrounding a healthy person’s nose and mouth, from which most of the air is drawn into the lungs [62]. These surfaces were used to calculate the probability of an aerosol encounter, which was used as a metric to compare the performances of different duct configurations. To apply the breath source, we used *CreateBaffles* utility in *OpenFOAM*. The upper part of Figure 2 shows the uniformly structured elements for the course, fine, and fine meshes. Fixed-value porous and fixed-flux pressure conditions were imposed on the supply air ducts. The atmospheric value and inlet-outlet conditions were employed to model the exhaust and recirculated air ducts, respectively. Based on the DIN EN-14750-1:2006 standard [63], the vehicle classification for urban and suburban rolling stock was divided into two categories: A and B. In type A, the standing members were higher than 4 passengers/m^2^; for category B, this value was lower or equal to 4 passengers/m^2^. The DIN EN-14750-1:2006 standard notes that the minimum fresh air rate for categories A and B are 14 and 10 hr^−1^, respectively. Furthermore, the Ashrae standard [64] determined that the air quality inside urban subway should be kept within the ranges of 10 to 13 hr^−1^ to provide adequate protection for public health. In this paper, category B satisfied our passengers’ configurations. In this study, the air change rate (ACR) of the urban cabin was 10 hr^−1^. The temperature values of the supply air, human breath, ambiance, and other cabin interior surfaces (such as mannequins, seats, and walls) were set at 20 °C, 30 °C, 25 °C, and 27 °C, respectively. The supply temperature was selected based on the standard ambient temperature for summer season. The temperature of a healthy human being is approximately 37 °C, while the exhaled breath temperature varies between 30 °C to 34 °C [65]; we set the human breath temperature to the lower temperature (30 °C) of this range to consider a lower evaporation of aerosols. The ambiance was set to the standard temperature and pressure (STP) conditions, and the thermally active surface temperature was set at 27 °C [66]. We defined thermally active surfaces in various HVAC systems as surfaces that are in direct contact with people or their respiratory flows. Figure 3 shows different views of the mesh configuration. Figure 4 shows mannequin meshing, which uses the snappyHexMesh utility in *OpenFOAM*. Figure 5 shows the schematic of the six cases. Red, yellow, and green mannequins represent infected individuals standing or sitting at the center (under combined exhaust and fresh air ducts), under the fresh air ducts, and under the recirculated ducts of the subway, respectively. The cell sizes of the mouth domain and ventilation ducts were 2 × 10^−3^ m and 5 × 10^−2^ m, respectively. The main input characteristics of the evaluated case studies are demonstrated in Table 1.

In this numerical work, we considered the sedentary activity for the external human body with no movement inside the urban subway. The value of clothing insulation was set at 0.60 clo. The *Clo* is a non-systematic unit for clothing insulation, determined as 0.155 °Cm^2^/W [67,68]. In other words, we supposed that mannequins’ clothing level is trousers with a long-sleeve shirt. In this simulation, we used the fixed temperature boundary condition over the mannequins selected from Angelova’s work [69].

### 2.3. Numerical Technique and Boundary Conditions

**Figure 6:**
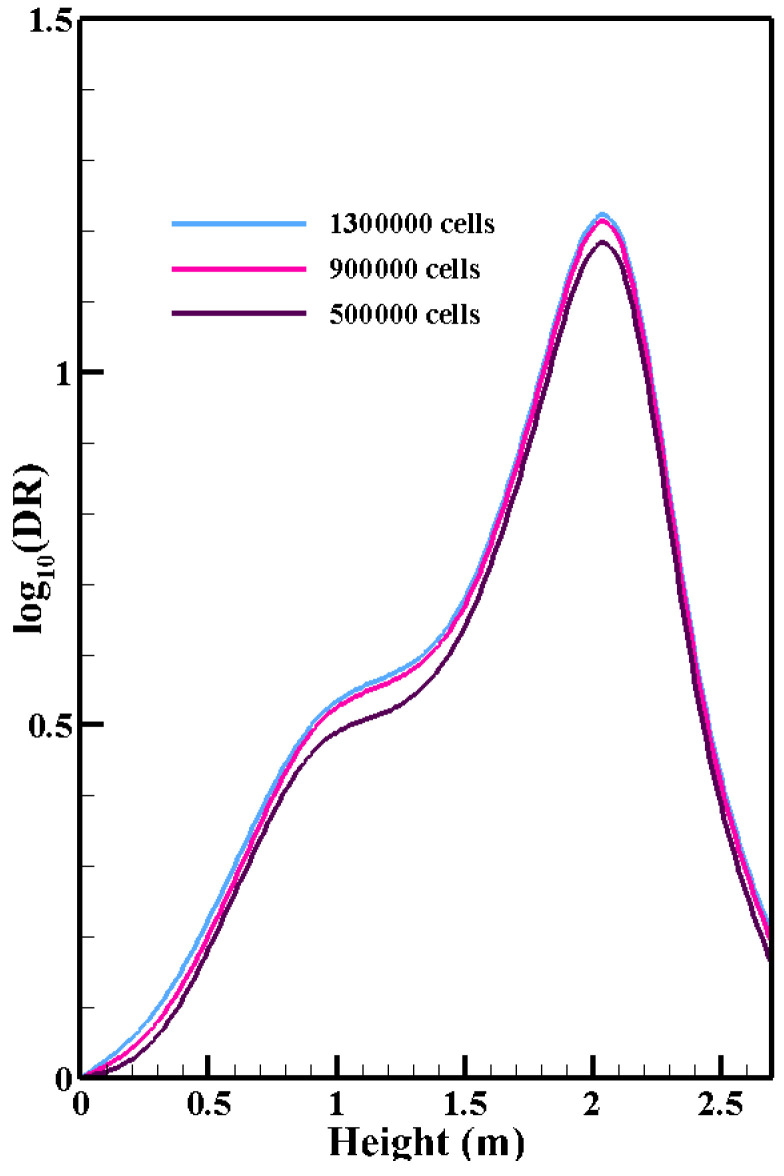
Log_10_(DR) at 20 cm in front of mouth vs position along the height of urban subway obtained using coarse mesh 500,000, fine mesh 900,000, and finest mesh 1,300,000 (the structures of these meshes are shown in Figure 3). *DR* is the ratio of the continuous breathing source concentration to the initial concentration in the subway cabin.

Table 2 compares our data with the experimental visualizations presented by Tao et al. for the 3D numerical simulation of the discharge velocity of the supply air [56]. The supply air of the urban subway passes through a porous media. The equation for the pressure drop through the porous media at the supply ducts is:

(12) $$\Delta p=\frac{1}{2}C_{2}\rho \Delta nv^{2}+\frac{\mu }{\alpha }\Delta nv,$$

where μ is the aerodynamic viscosity, which is set at 1.8 × 10^−5^; v is the air velocity; and Δn represents the thickness of the supply panel. To obtain accurate results, a porous boundary condition was applied to the supply air ducts. The values of $C_{2}$ and $\frac{1}{\alpha }$ were set at 150,000 m^−1^ and 1.5 × 10^6^ m^−2^, respectively. The calculated points were positioned at the two ends and in the middle of the subway cabin and averaged in the z-direction. We calculated the average velocity and temperature in the passenger compartment of the subway at three horizontal measurement points and compared the findings with those from the experimental work of Tao et al. (*v_a_* and *T_a_*) [56]. Additionally, the maximum differences in the horizontal (*v_x_* and *T_x_*) and vertical (*v_z_* and *T_z_*) velocities and temperatures were calculated and compared. The maximum relative deviations of the velocity and temperature for the two cases in Table 2 were approximately 1.2% and 1.79%, respectively. The maximum temperature and velocity differences were used as evaluation metrics for the air distribution performance. These values were calculated using specific points along the x- and z-directions of the subway cabin. The European norm DIN EN-14750-1:2006 standard was applied to calculate the temperature and velocity differences and define specific points [63]. In addition, a new index, namely, the aerosol encounter probability inside an urban subway was defined to quantitatively evaluate the degree of the suppression effect of the HVAC system on airborne transmission. A grid independence test was performed to compute the required number of numerical cells to obtain convergent results. To obtain grid-independent results, simulations were performed on three different mesh topologies. A numerical grid with 900,000 cells for one core of the parallel solution was selected which had a convergent solution with an optimized computational cost (Figure 6). The maximum skewness of the grid was 0.3, which was suitable for obtaining accurate results. The number of grid points over the mannequin in the fine mesh was 8000. The Y^+^ values were well below 1.0 over most regions of the mannequin surface and were limited to a maximum of 3.0, and the maximum value of x^+^ and z^+^ on any part of the mannequin was 2.0. The mesh quality influences the detailed flow in the vicinity of the complex geometry. However, as shown in our mesh independent test, selection of a suitable wall function near the wall limits the influence of mesh conditions on aerosol behavior. In addition, we selected a time step of 10^−6^ s to obtain an appropriate and convergent solution. The time-step size was set based on a maximum Courant number of 10. The results show that both the standard *k–ε* and RNG *k–ε* models can accurately simulate the fluid flow and temperature inside an urban subway HVAC system. The RSM turbulent approach had similar results to *k-ε* for the velocity parameter, but for temperature fields, the RSM results were significantly closer to Tao et al.’s study [56]. The results of the k-ε model were closer to the experimental data. A comparison of the four turbulence models is presented in Table 2. In addition, the standard wall function was applied to predict the near-wall turbulence. A no-slip boundary condition was applied to all solid surfaces (except the vents). The maximum velocity rate at the supply duct inlet was 4.2 m·s^−1^, and recirculated air and exhaust ducts circulated 75% of aerosols exiting through the HVAC system. A *ZeroGradient* boundary condition was applied at the exhaust ducts.

### 2.4. Modelling of Aerosol

The temporal average was used to investigate the urban subway flow structure and is defined as [29]

(13) $$\varphi =\frac{1}{t_{2}-t_{1}}\int_{t_{1}}^{t_{2}}\varphi dt,$$

where *t_1_* is the time in which all aerosols reach a dynamic balance in the simulation, and *t_2_* is selected based on the traveling time of the urban subway between stations. The diffusion of aerosols from the infected person’s mouth to the subway domain occurs close to equilibrium conditions only if the environmental concentration is minimal. Nonequilibrium diffusion occurs in environmental flows with high concentrations. A healthy individual inside an urban subway may create such environmental flows. We used a suitable initial condition to simulate aerosol transport to determine if all aerosols reached a dynamic balance. The equation $DR=\frac{C}{C_{0}}$, which is used in the probability of an aerosol encounter, is the ratio of continuous breathing source concentration to initial concentration in the subway cabin. The initial concentration cannot be equal to zero. The minimal concentration (equilibrium flow) value should be used to start the simulation. If we solved it with a higher concentration, the problem would not be related to the present work; it would be related to the science of aerosol interaction (nonequilibrium flow). Some researchers have used appropriate initial conditions in their simulations to ensure an equilibrium flow field and obtained satisfactory results [70,71]. Based on the typical temperature and ventilation velocity in an urban subway, the Wells–Riley equation,$P=1-e^{\frac{-qt}{DR}}$, may be used for aerosol encounter probability, where q is the quantum generation rate for an infected person (quanta·s^−1^) [49]. A higher DR value in a certain zone indicates a higher risk of transmission [28]. The number of ejected particles is defined [36] as

(14) $$N=\sum_{0}^{t}C\dot{V},$$

where t and $\dot{V}$ are the exposure time and human breathing rate, assumed in this study to be equal to 180 s and 0.00033 m^3^·s^−1^, respectively [36]. In this study, we assumed continuous flow at the breathing sources inside the cabin. Besides, we assumed that the air at the individuals’ mouths is constantly outward at a breathing frequency of 0.2 breaths/s (respiratory period of ~5 s and 12 breathing per minute). The simulation time is sufficiently long; such periodic effects can be neglected because our focus is on airborne transmission far from the emission source. The continuous breathing model is suitable for analyzing virus movement inside an urban subway cabin over exposure time. Therefore, the pulsatile nature of breathing was neglected. Instead, we used a steady flow velocity that was equivalent to the average velocity of the entire respiratory period. This length of time is mandatory for an exhalation-based viral load and essential for simulating aerosol dispersion in a specific confined space. Some researchers used this assumption (continuous flow at the breathing sources) to simulate aerosol extraction by breathing and achieved satisfactory results under flow settings similar to our current configuration [72,73]. The modeled breathing source had a hydraulic diameter of 0.04 m.

Experimental studies have shown that coughing and sneezing have a higher risk of causing infection owing to the intensity of the created particles [74]. However, sneezing or coughing was not simulated in this study. To date, no study has determined the number of viral particles required to infect an individual. Therefore, the number of particle encounters required to get infected was assumed to be 50, based on the studies by Zhang et al. [36] and Kolinski and Schneider [75]. To simulate the breath source, a source inside an urban subway cabin was applied for each case. Fixed flux and constant value conditions were used for velocity and concentration at the source, respectively. The variation in aerosol concentration with temperature was considered in this study. Given the complex velocity distributions and temperature gradients inside a cabin, averaging the temperature and velocity values is essential. The present analysis considered thermal gradients by solving the energy equation to refine the results. Solving the energy equation can influence the second term of the Navier–Stokes equation (Equation (2)/$g\cdot x\nabla (\frac{\rho }{\rho_{0}})$).

The density directly affects the aerosol behavior under gravitational force. Gravitational force is an important parameter that determines the traveling direction of the aerosol cloud inside the urban subway cabin, similar to buoyancy. Bhardwaj and Agrawal [76] introduced the diffusion coefficient as a function of environmental temperature as follows:

(15) $$D_{eff}=2.5\times 10^{-4}\exp\overset{}{}(-\frac{684.15}{T}).$$

The energy equation was solved to determine the thermal gradient and its effects on the aerosol motion to track aerosol clouds in the context of diffusion through the urban subway. The thermal gradients affect the density, hence, the gravitational force. The interplay between gravitational force, Stokes drag force, and Brownian motion affects the behavior of an aerosol cloud inside an urban subway. The induced ventilation force consists of the mean flow velocity, turbulent fluctuation, mean aerosol velocity, and fluctuating aerosol velocity effects [29]. The sum of these four effects is known as the Stokes drag force [18,29,77]. Moreover, because a buoyant solver was applied, the effect of Brownian motion was ignored in this study. Therefore, the aerosols resulting from continuous breathing were assumed to be transported by induced ventilation and gravitational forces.

## 3. Results and Discussion

**Figure 7:**
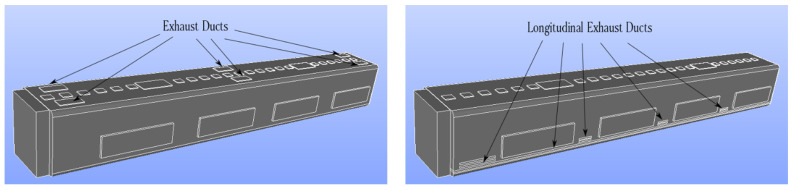
Comparison between a conventional (**left side**) and the proposed (**right side**) HVAC systems for an urban subway cabin.

The spread of particles (such as SARS-CoV-2-containing aerosols) increases when the infected individual breathes near the supply air ducts. In this section, we describe the concentration contours for six distinct breathing sources at different exposure times. A significant feature of subway transport is the regular stopping of subway trains for passenger boarding and disembarking, further increasing the risk of viral transmission. The proposed HVAC system for urban subways helps to reduce suspended aerosols in the cabin and mitigates the risk of viral transmission. The distance between the stations of the urban subway considerably affected the exposure time. A 3D schematic of the proposed HVAC system is depicted on the right side of Figure 7. According to this figure, the only difference between a conventional subway HVAC system (left side of Figure 7) and the proposed system is the application of a longitudinal exhaust duct along the cabin floor, instead of the ceiling, to reduce aerosol concentration.

**Figure 8:**
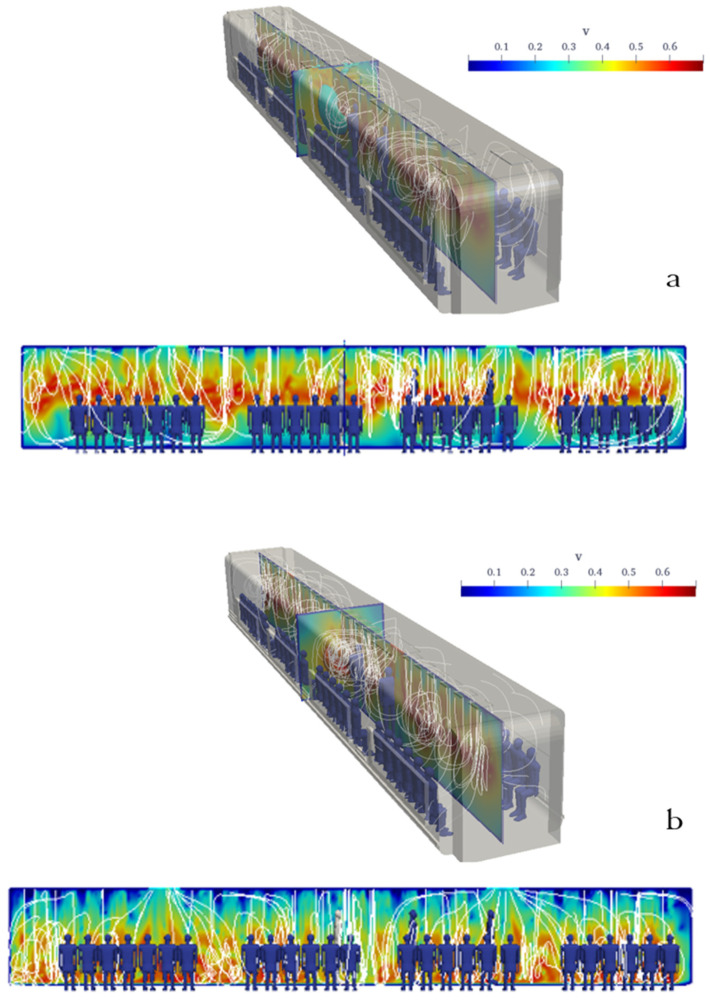
Comparison between the streamlines of a conventional (**a**) and the proposed (**b**) urban subway HVAC systems. The velocity contours shown are in the mid-planes.

Figure 7 shows a schematic of the computational domain, including the supply air ducts, exhaust ducts, and recirculated air ducts. In Figure 8, the airflow patterns of the conventional and proposed HVAC systems are compared. To compare the two HVAC systems, the areas of the exhaust ducts were made equal. As shown in Figure 8, the airflow of the proposed HVAC system was mostly downward, which did not permit the aerosols to spread horizontally.

### 3.1. Infected Individual Breathing near the Supply and Exhaust Ducts (Cases 1 and 2)

**Figure 9:**
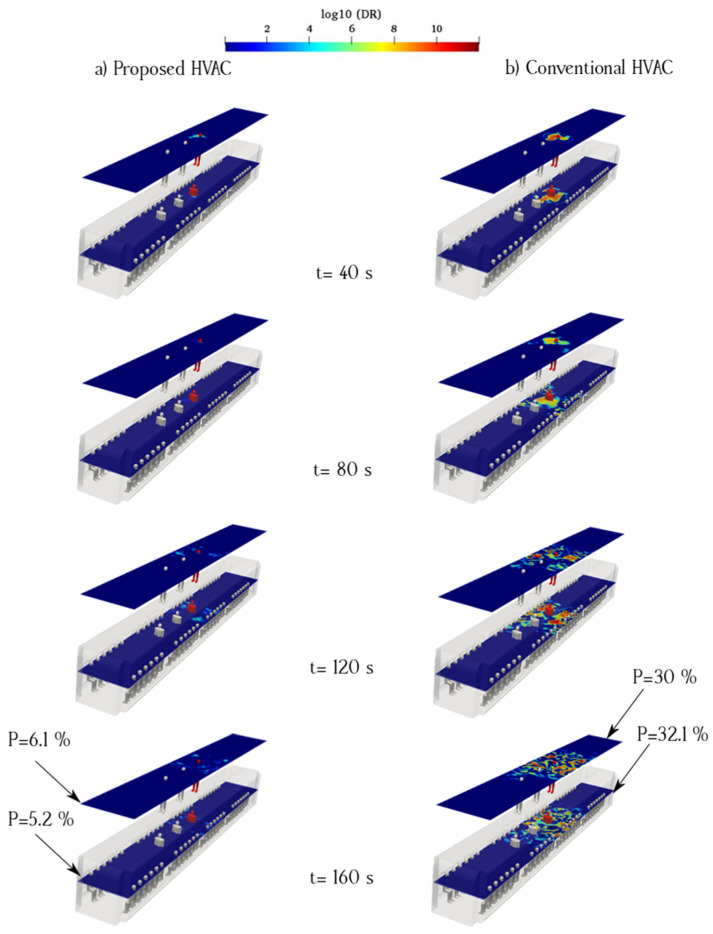
Time evolution of the counters of log_10_(DR) when the infected individual was standing near the supply and recirculated air ducts of the proposed (**a**) and conventional (**b**) HVAC systems (Case 1).

**Figure 10:**
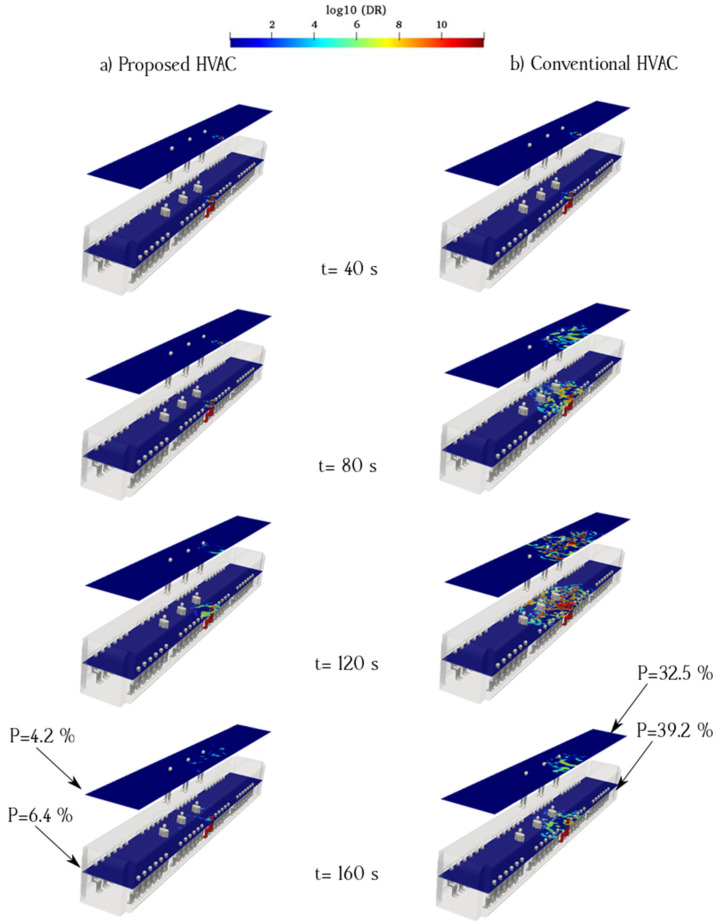
Time evolution of the counters of log_10_(DR) when the infected individual was sitting near the supply air and recirculated ducts of the proposed (**a**) and conventional (**b**) HVAC systems (Case 2).

The flow pattern is complex at the center of an urban subway cabin because of the upward flow toward the exhaust ducts and downward flow of fresh air induced by the porous boundary. When an infected individual breathes near the exhaust ducts, the virus can readily exit the cabin. However, the downward flow of fresh air disperses aerosols to a certain extent. The flow pattern around the exhaust duct was not smooth, causing a more complex flow around the aerosol cloud. The breathing flow was much weaker than the ventilation flow; therefore, the ventilation flow can remove exhaled aerosols from the infected passenger. Figure 9 and Figure 10 show the counters of log_10_(DR) when the infected individual was standing and sitting, respectively (Cases 1 and 2). The left column (a) shows the proposed HVAC system, and the right column (b) shows the conventional HVAC system of the urban subway. As shown in Figure 9, when the infected individual was standing and breathing near the exhaust and fresh-air ducts at the center of the cabin, the viral load was maintained than that of the proposed HVAC system over time. In the proposed HVAC system, the aerosol concentration near the floor of the subway cabin was, at times, higher than that of the conventional system.

To better understand the behavior of aerosols inside the urban subway, the average value of log_10_(DR) along the sampling surfaces is shown in Figure 9 and Figure 10. Logarithmic scales were used to delineate concentration figures. As shown in Figure 9, the average aerosol concentration along the standing sampling surface of the proposed HVAC system was lower than that of the conventional HVAC system. In addition, the average aerosol concentration along the sitting sampling surface for the proposed HVAC system was much lower than that of the conventional HVAC system. The main difference between the proposed HVAC system and conventional HVAC system was the flow pattern inside the urban subway. The longitudinal ventilation flows of the conventional HVAC system dispersed the aerosols more than those of the proposed HVAC system. The probability of aerosol encounters over the sampling surfaces for Case 1 is shown in Figure 9. The highest value of the aerosol encounter probability corresponding to the sitting sampling surface of the conventional HVAC system was 32.1%. This rate was 83.8% higher than that for the same surface in the proposed HVAC system.

Figure 10 shows the time evolution of the counters of log_10_(DR) for case 2, in which the infected individual was sitting near the exhaust and fresh-air ducts. The left column shows that in the case of the proposed system, a higher proportion of the aerosol cloud moved toward the cabin floor. The remaining part was dispersed in the surrounding spaces following the ventilation flows. However, in the case of the conventional HVAC system, aerosol clouds dispersed upward and affected a more extensive zone. The right side of the cabin, where the infected passenger was seated, contained a higher concentration of aerosols in both the HVAC systems. The aerosol concentrations in the standing/sitting surfaces of the conventional HVAC system were higher than those on the same surfaces in the proposed model. The probability of aerosol encounters at each sampling surface in Case 2 is shown in Figure 4. In the conventional model, the maximum aerosol encounter probability was 39.2% for the sitting sampling surface. This rate was 6.125 times higher than that of the proposed model. The lowest rates were on the surface in both models, and the rate for the proposed HVAC system was significantly lower, i.e., 4.2%. Impeding aerosols from rising inside a subway cabin is crucial to reduce the probability of aerosol encounters. By locating exhaust ducts near the floor of the subway, the overall aerosol concentration can be reduced through the suppression effect, which is defined as the rapid removal of indoor air pollutants by ventilation in a short pathway. This concept was considered in the development of the proposed model. However, some amount of aerosol continues to rise within the proposed HVAC system owing to the velocity distribution irregularities and position of the recirculated air ducts along the ceiling.

Different ventilation arrangements alter the flow circulation and therefore influence aerosol dispersion. The airflow circulation inside a subway cabin stems from the porous boundary of the HVAC system and secondary induced flow. Therefore, given a fixed position for the passengers, the smooth airflow part under the fresh-air ducts is an appropriate location for standing and sitting, whereas near the exhaust ducts is an inappropriate location. However, the movement of passengers inside the cabin may perturb the airflow pattern and disperse particles, thus increasing the risk of transmission.

The entering airflow pattern of an urban subway HVAC system depends on the porosity parameters. The interaction between the entering and exiting airflows with recirculated air ducts at the center of the urban subway increases the irregularity of the flow pattern at the aforementioned locations. The proposed HVAC system can suppress breath particles at the center of the urban subway HVAC system. In the proposed model, the concentration of particles and area of their spread were lower than those in a conventional HVAC system.

The conventional subway HVAC system can readily spread viruses in the top part of the subway cabin owing to its continuous flow and inconvenient exhaust duct configuration. For example, when an infected individual breathes near the supply air ducts, respiratory particles spread through the airflow, and a healthy standing passenger near the exhaust ducts can be infected.

### 3.2. Infected Individual Breathing near the Fresh-Air Ducts (Cases 3 and 4)

**Figure 11:**
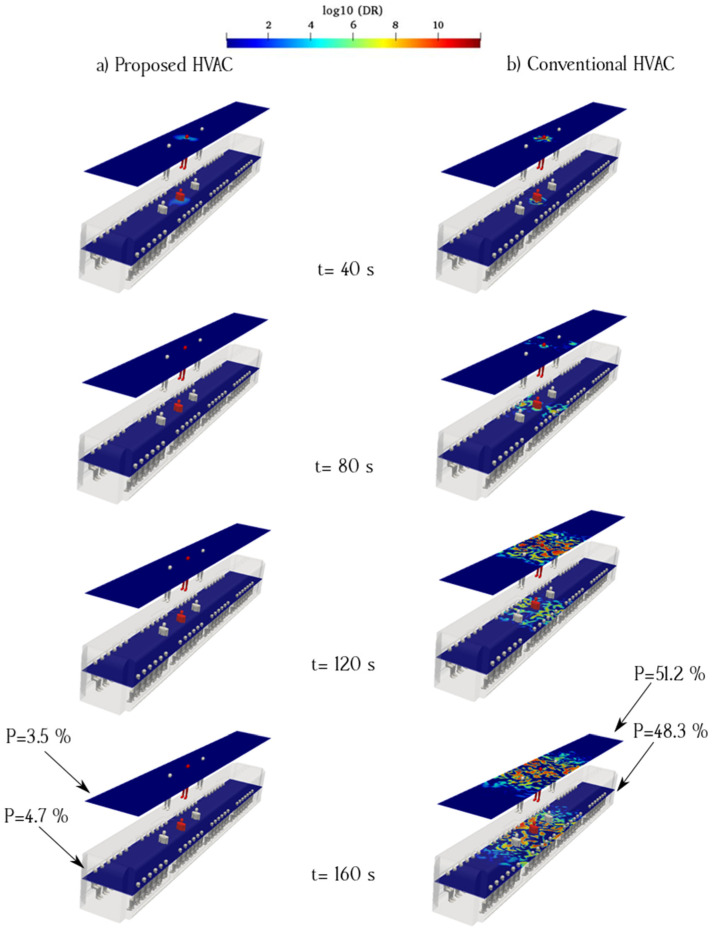
Time evolution of the counters of log_10_(DR) when the infected individual is standing near the supply air ducts of the proposed (**a**) and conventional (**b**) HVAC systems (Case 3).

**Figure 12:**
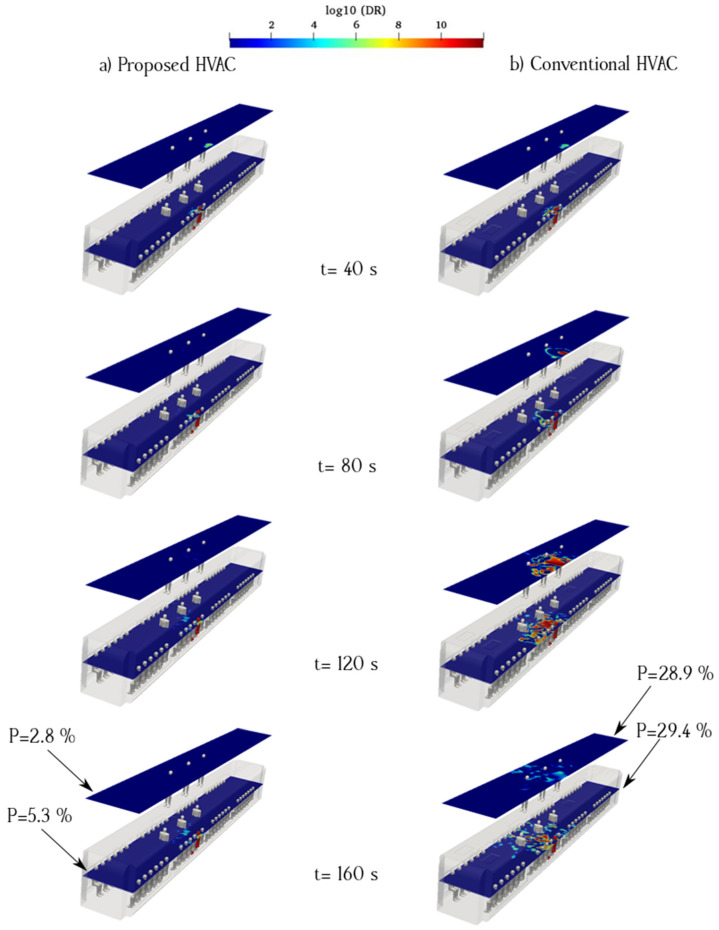
Time evolution of the counters of log_10_(DR) when the infected individual is sitting near the supply air ducts of the proposed (**a**) and conventional (**b**) HVAC systems (Case 4).

Aerosol dispersion inside an urban subway depends on the airflow pattern of the HVAC system. When an infected individual breathes near the supply ducts, viral particles take the longest path before being removed by the exhaust ducts. In this case, the viral particles remain suspended for an extended period, increasing the risk of infection (Figure 11 and Figure 12). This risk is further increased if the infected person remains in the subway cabin for a longer time during the boarding and disembarking of passengers. In this case, we assumed that the infected individual and healthy individuals remain in a fixed location during the subway movement. By determining the safe zones and sitting on seats in those zones in the urban subway, the risk of transmission can be minimized. The crucial protective effect of face masks against SARS-CoV-2 has been highlighted by scientific authorities [18,20,57]. People inside the urban subway should wear face masks and sit on seats with appropriate social distancing. Airflow within the urban subway carries aerosols from breath source(s), which are then expelled from recirculated air ducts or atmospheric exhaust ducts. Furthermore, the application of HEPA filters and ultraviolet light emitters inside the recirculated air ducts decreased the virus concentration. In reality, aerosol movement resulting from ventilation flows may behave like a cloud flow, especially at small shear stresses of fresh air. The conventional HVAC system gives rise to higher log_10_ (DR) values than the proposed HVAC system. The distribution patterns of breath aerosols demonstrate that when an infected individual breathes under the fresh-air ducts of the cabin, the conventional HVAC system airflow carries these particles to the exhaust ducts. In this case, the traveling aerosol pathways are near the standing sampling surface and traverse a long distance toward the exhaust ducts, thus increasing the aerosol encounter probability.

Figure 12 shows the effect of the HVAC system type on aerosol dispersion for an infected passenger seated under a fresh-air ducts. With the conventional HVAC system configuration, the results show that aerosols spend a long time in suspended condition before reaching the exhaust duct when the infected person sits under the fresh-air duct. The aerosol concentration on the same side of the cabin as that of the infected passenger was higher than that of the other side. In contrast, for the proposed HVAC system, the aerosol concentration is considerably lower, given that exhaust ducts near the floor of the cabin can quickly remove aerosols and suppress suspended aerosols to reduce the spread of particles inside the cabin. Figure 12a shows the aerosol concentration counters when an infected individual was seated and breathing near the supply ducts in the proposed model. The aerosol path to the longitudinal exhaust ducts was narrow and continuous. Figure 11a shows the aerosol concentration for an infected individual standing and breathing continuously near the supply ducts in the proposed model, assuming a location similar to that in the sitting case. In this case, the flow pattern of aerosols is narrow and linear, which indicates that the aerosol spread mitigation efficiency is higher than that of the conventional HVAC system.

### 3.3. Infected Individual Breathes near the Recirculated Ducts (Cases 5 and 6)

**Figure 13:**
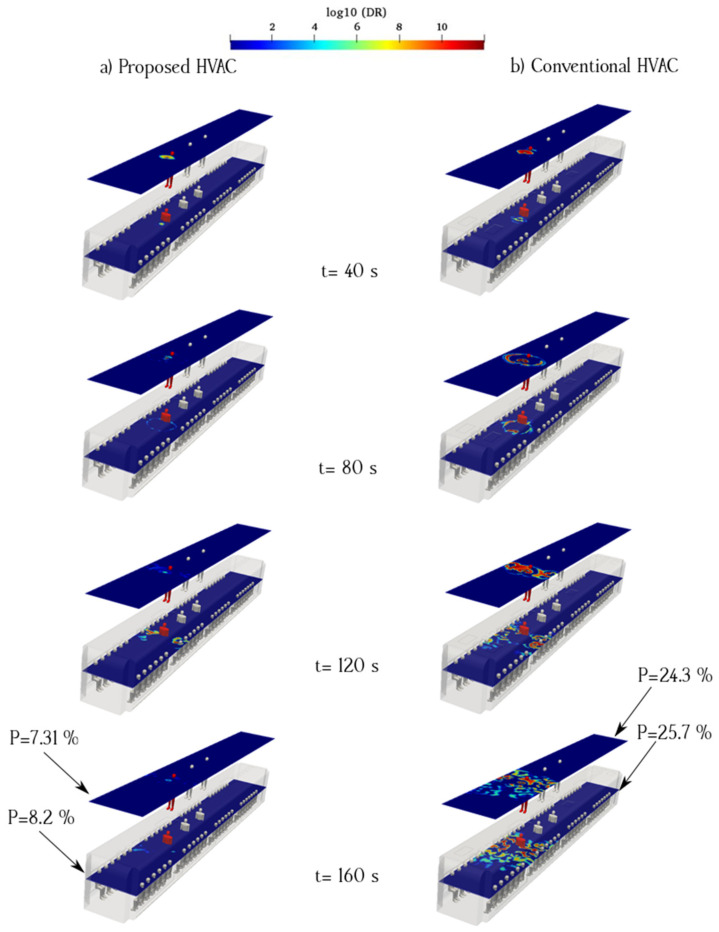
Time evolution of the counters of log_10_(DR) when the infected individual is standing near the recirculated ducts of the proposed (**a**) and conventional (**b**) HVAC systems (Case 5).

**Figure 14:**
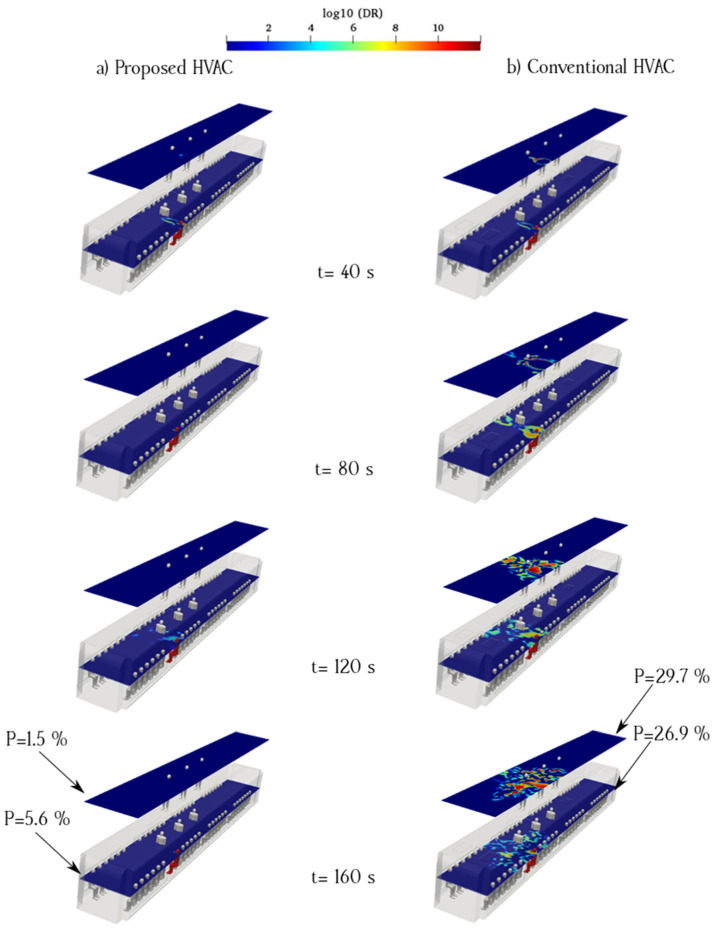
Time evolution of the counters of log_10_(DR) when the infected individual is sitting near the recirculated ducts of the proposed (**a**) and conventional (**b**) HVAC systems (Case 6).

Figure 13 and Figure 14 show the aerosol concentrations in cases 5 and 6, in which the infected individual is standing and sitting near the recirculated air ducts, respectively. By comparing the log_10_(DR) counters of cases 5 and 6, we can conclude that a uniform air distribution reduces the suspension of aerosols under the recirculated and return ducts inside the subway cabin. The horizontal spreading of aerosols was minimal in the proposed model. The proposed HVAC system results in a lower aerosol concentration than that of the conventional model if the infected individual is in a standing position. The highest probability of aerosol encounters in the proposed HVAC system was 8.2% for the sitting sampling surface, which was 68.09% lower than that of the conventional HVAC system (Figure 13). Based on Figure 13, the highest aerosol encounter probabilities for the seated case near the recirculated ducts in the proposed and conventional HVAC systems were 8.2% and 25.7%, respectively. Our study shows that conventional HVAC systems can propagate airborne virus-bearing aerosols near recirculated ducts on the top side of the cabin, which can increase the transmission risk. In other words, the infected person may stand under the exhaust ducts, and the flow pattern expels particles in the conventional HVAC system. The log_10_(DR) variations over the sampling surfaces for Cases 5 and 6 are shown in Figure 13 and Figure 14, respectively.

### 3.4. General Comparison of Cases 1 to 6

**Figure 15:**
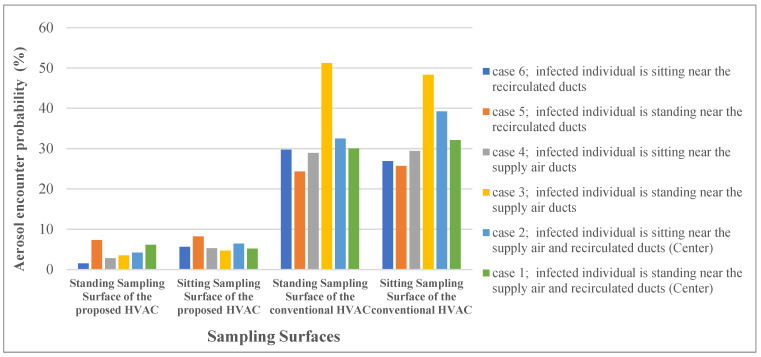
Comparison of aerosol encounter probability for cases 1 to 6.

Figure 15 summarizes the aerosol encounter probabilities (%) from the aforementioned simulated cases. The y-axis indicates the probability of an aerosol encounter, and the x-axis shows the sampling surfaces of both conventional and proposed HVAC systems. The vertical distribution of aerosols is shown for each case and can be used to understand the transmission risk of each sampling surface. The aerosol encounter probability is an index that can determine the exhaled aerosol behavior inside an urban subway. The values shown in Figure 9 are temporally averaged. The aerosol dynamics were plotted only for points along the sampling surfaces; otherwise, the values were set to 0. The spatial values of the surfaces were calculated for each time point. The average time for aerosol encounter probability was then calculated. The aerosol encounter probability at the standing surface of the urban subway for each HVAC model was higher than those for the other surfaces. This implies that when an infected person stands inside an urban subway cabin, the risk of aerosol transmission increases. The highest probability of aerosol encounter for the two sampling surfaces corresponds to the conventional HVAC system in Case 3. In this case, the infected individual stands under the cabin’s fresh-air duct, where the incoming air flows with small shear stress and spreads the aerosol throughout the cabin toward the exhaust ducts. Irregularities in the velocity distribution caused by porous boundary conditions intensify the risk of transmission. However, for the proposed HVAC system, exhaust ducts were installed near the cabin floor, which could transfer aerosols via a short and safe pathway. Overall, the proposed HVAC system was more efficient in the rapid removal of aerosols through a safe pathway. The maximum viral transmission probability in the proposed HVAC system occurred in Case 5, with a rate of 8.2%, which was 68% less than that under similar conditions in the conventional model. In other words, by applying the introduced HVAC system to urban subways, the mean values of the aerosol encounter probability decreased by approximately 84% compared to those of the conventional system.

An important result of this study was the determination of the transmission pathway of aerosols inside urban subways and the corresponding hazard zones (i.e., the space with higher probability of infection) under conventional subway HVAC settings. Assuming that an ample space of the urban subway is under fresh air injection, aerosols can spread both vertically and horizontally inside the conventional cabin. Based on our results, aerosols can move along a conventional urban subway and then rise to exit from the exhaust and recirculated ducts. During that time, healthy passengers who stand or sit near the exhaust and recirculated ducts are more vulnerable to viral particles compared to other locations. The unsafe zones of conventional urban subways with a higher probability of aerosol encounters can be calculated using equation (16) [28]

(16) $$P_{tot}=\frac{\sum P_{unsafe,i}\times S_{unsafe,i}}{S_{tot}}$$

where $P_{tot}$, $S_{tot}$, $P_{unsafe,i}$ and $S_{unsafe,i}$ are the total probability of aerosol encounter, total sampling surface, unsafe probability of aerosol encounter of each case, and unsafe area of each case, respectively. Using Equation (16) and passenger breathing zones, unsafe areas (defined as zones with high aerosol concentration [28], high aerosol retention time [29], and high vertical aerosol spreading) for each source of breathing were determined. Notably, the region near the recirculated duct yielded a significantly higher probability of aerosol encounters. A healthy person standing in a hot spot under the recirculated duct is exposed to several times higher aerosol concentration than a person in a safe area (under fresh air ducts). The proposed HVAC system for an urban subway can eliminate unsafe hot spots existing in the conventional HVAC system by effectively minimizing the aerosol travel time and path lengths when those are being extracted by the ventilation system.

### 3.5. Effect of Supply Temperature of HVAC for Case 3

**Figure 16:**
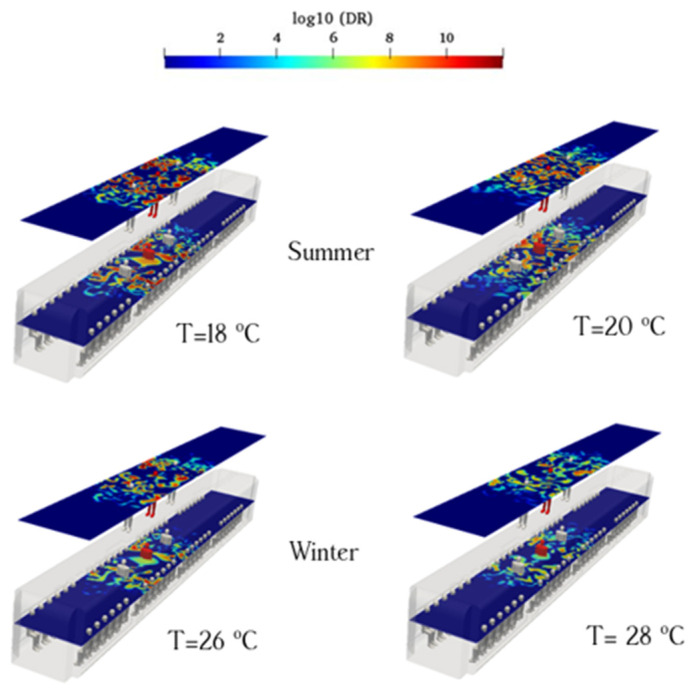
Evolution of the counters of log_10_(DR) when the infected individual is standing near the supply air ducts of the conventional HVAC systems (Case 3) at the time of 180 s for various temperatures (summer and winter season).

Based on the above analysis, when an infected individual is standing near the supply air ducts, the probability of aerosol encountering is high. In this study, we assumed four temperatures (18 °C, 20 °C, 26 °C, and 28 °C) to better understand aerosol transmission inside urban subways and calculate aerosol encounter probabilities. The present study considered the effects of temperature on aerosol behavior. The supply air temperature differed between winter and summer, with a higher thermal gradient in winter. The higher temperature of the induced air supply in winter leads to higher interaction between the thermal gradient and aerosol clouds. Under these conditions, the surficial evaporation of aerosol clouds inside urban subways decreases the probability of aerosol encounters. The aerosol concentrations over the sampling surfaces at various temperatures are shown in Figure 16. The calculated probabilities of aerosol encounter of standing sampling surface for 18 °C, 20 °C, 26 °C, and 28 °C temperatures of supply ducts were 55.32%, 51.2%, 39.2%, and 30.1%, respectively; the values for sitting sampling surface were 49.89%, 48.3%, 40.2%, and 33.2%, respectively. The interplay between the continuous temperature gradient of the induced air supply and buoyancy affects the aerosol cloud diffusion process inside the urban subway. The larger the difference between the ambient air and respiratory flow (containing aerosol cloud), the higher the buoyancy of the respiratory flow and lower the lateral dispersion of aerosols. The increase in aerosol-cloud buoyancy decreases virus transmission owing to the interaction between up-direction buoyant flow and down-direction shear flow of fresh air. The interaction between aerosol clouds and ambient air is an important mechanism for decreasing viral transmission. These results are consistent with those of He et al. [39], Nazari et al. [18], and Rezaei et al. [35]. At lower temperatures of the induced air flow, the effects of the mentioned aerosol buoyancy decrease, which can help disperse the viruses more with the induced air supply. We discuss the importance of this study from the perspective of reducing the aerosol encounter probability inside urban subways using HVAC system duct configuration, supply air temperature, and ACR. Breathing aerosol lifetime is an important parameter that reflects the duration over which a healthy individual can get infected if one encounters an induced aerosol.

### 3.6. Effect of Supply Air change Rate (ACR) of HVAC for Case 3

**Figure 17:**
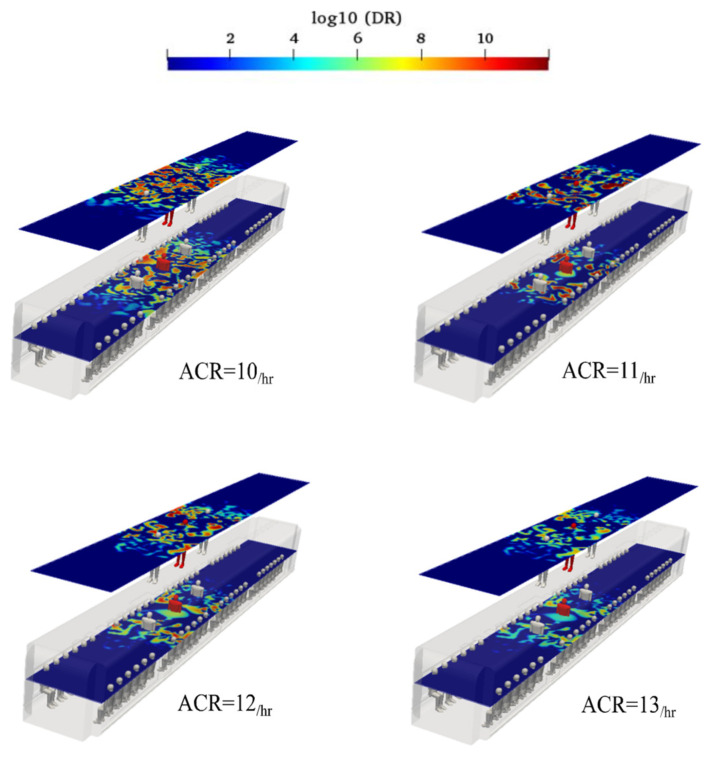
Evolution of the counters of log_10_(DR) when the infected individual was standing near the supply air ducts of the conventional HVAC systems (Case 3) at the time of 180 s for various well-ventilated air change rates.

This section investigates the effect of fresh air ACR inside urban subways on aerosol spread for case 3. Four ACRs were selected, and the aerosol encounter probabilities were calculated. To quantify the ACR effect, simulations were conducted using ACR values of 10, 11, 12, and 13 hr^−1^. Based on Figure 11, an ACR of 10 hr^−1^ with an induced temperature of 20 °C represents the worst-case scenario. In this case, aerosol propagation on both sampling surfaces was higher than that on the same surfaces in the conventional HVAC system. An essential method to reduce aerosol encounter is to increase the fresh air velocity, which can be achieved by increasing the ACR. The HVAC system consumes higher energy with an increase in the ACR value. The calculated probabilities of the standing sampling surface aerosol encounters for 10, 11, 12, and 13 hr^−1^ were 51.2%, 42.3%, 35.2%, and 30.1%, respectively; the corresponding values for sitting sampling surface were 48.3%, 40.9%, 30.5%, and 25.4%, respectively. The aerosol concentrations over the sampling surfaces for the various ACRs are shown in Figure 17.

**Figure 18:**
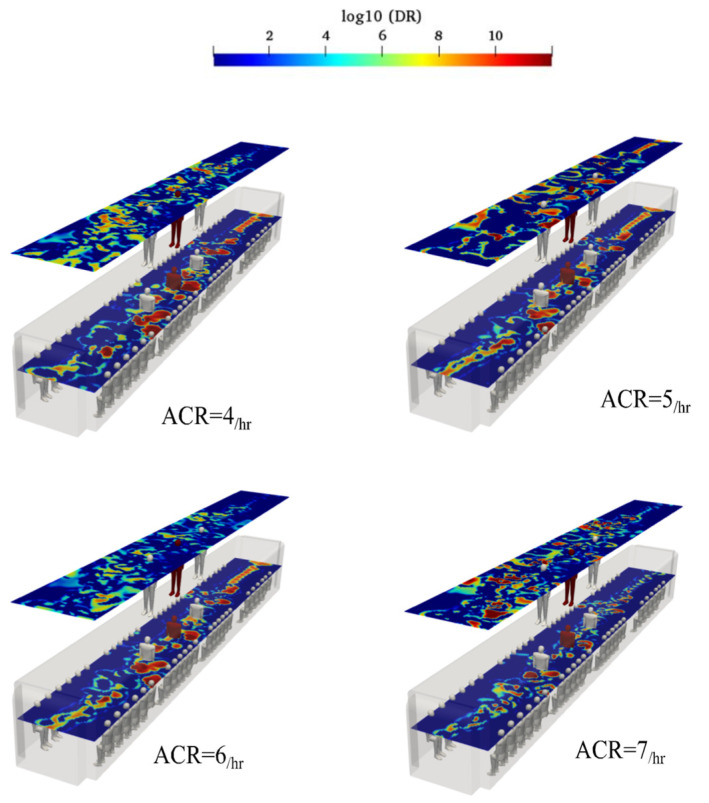
Evolution of the counters of log_10_(DR) when the infected individual was standing near the supply air ducts of the conventional HVAC systems (Case 3) at the time of 180 s for various poorly ventilated air change rates.

In some cases, the supply air ducts cannot inject appropriate amount of fresh air into the subway cabin. To consider this scenario, poorly ventilated ACRs were investigated. Figure 18 shows the effects of ACRs of 4, 5, 6, and 7 hr^−1^ with an induced temperature of 20 °C on the breathing aerosol spreading inside the cabin. In a poorly ventilated ACR, the weak shear flow cannot overcome the aerosol cloud to change its path and direct it toward the exhaust ducts. The zone with high concentrations of aerosol encounter levels were created in the poorly ventilated scenario, which corresponded to the locations where virus transmission was greater than that in other places. These zones were spread in the longitudinal direction of the cabin, allowing the breathing aerosols to move in any desired direction. The calculated probabilities of the standing sampling surface aerosol encounters for 4, 5, 6, and 7 hr^−1^ were 76.1%, 74.4%, 68 %, and 65.1%, respectively; the corresponding values for sitting sampling surface were 71%, 70.2%, 62.2%, and 60.3%, respectively.

### 3.7. Effect of Imperfect Filtration of HVAC for Case 3

**Figure 19:**
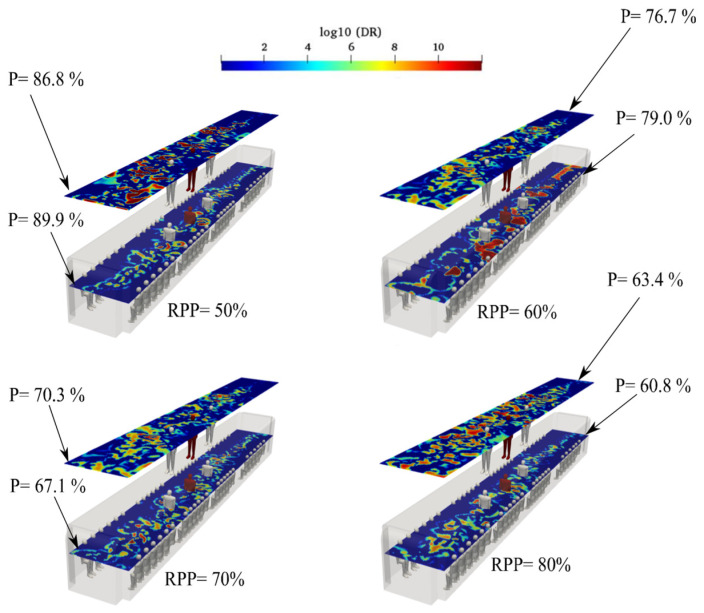
Evolution of the counters of log_10_(DR) when the infected individual was standing near the supply air ducts of the conventional HVAC systems (Case 3) with imperfect filtration at the time of 180 s for various removed particle present (RPP).

In all the sections mentioned above, filters inside the ducts were assumed to eliminate all airborne viruses. To simplify the simulations, we ignored the conditions that lack HEPA filters and assumed that the aerosol concentration in the injected fresh air was near zero. With the constant use of filters over time, some features of these filters change and lose their effectiveness, and aerosols can penetrate the cabin. A common index of HEPA filters, namely, removed particle present (RPP), was used to rate the efficiency of the filters. In the HVAC system of an urban subway, fresh outdoor air flows through the supply air duct, combines with recirculated airflow, and then flows through the supply air duct to the cabin. Part of the indoor air returns to the exhaust air duct and then enters the outdoor atmosphere. Because recirculated air and exhaust ducts circulate 75% of aerosols exiting through the HVAC system, HEPA filters are placed in all ducts to clean the air entering the cabin. To quantify the RPP effect, simulations were conducted with the RPP values of 50, 60, 70, and 80, and corresponding aerosol encounter probabilities were calculated. Notably, the worst case (case 3) with imperfect filtration in a conventional HVAC system was selected. The probability of aerosol encounters at each sampling surface in Case 3, with imperfect filtration, is shown in Figure 19. The highest aerosol encounter probability for an RPP of 50% was 89.9%. When the air supply blows at a particle number of 20, exhaled air interacts with fresh air. The mixing of these concentrated flows intensifies aerosol transmission in the vertical direction of the cabin. The effect of imperfect filtration was studied by changing the concentration in the fresh air ducts based on the following equation:

(17) $$\mathrm{RPP}=(\frac{C_{\mathrm{beyond}}-C_{\mathrm{front}}}{C_{\mathrm{beyond}}})\times 100,$$

where $C_{\mathrm{beyond}}$ and $C_{\mathrm{front}}$ are the concentrations of particulate matter outside and in front of the filter, respectively.

## 4. Conclusions

In this study, we computationally investigated the effects of a proposed HVAC system on the spread of viruses, such as SARS-CoV-2 inside an urban subway system. We employed a transport equation for the concentration, which was added to the basic buoyant solver to resolve aerosol transmission inside the urban subway by considering the thermal, turbulent, and induced ventilation flow effects. The mechanisms of virus spread through the movement of virus-containing aerosols were investigated using the *OpenFOAM* C++ libraries. Our simulation approach was validated using experimental data by applying a porous boundary condition to fresh-air ducts. Subsequently, we used a numerical approach to investigate the airborne transmission pattern and the associated infection risk for different ventilation designs and source locations. The exhaust ducts of a conventional HVAC system are located along the cabin ceiling and near the fresh-air ducts. These configurations create longitudinal ventilation flows inside the cabin and remove aerosols along long paths. This study introduced aerosol encounter probability to represent the probability of infection during airborne transmission. An increase in the aerosol encounter probability owing to the flow pattern depends on the exhaust configuration. To suppress aerosol encounter probability, we changed the exhaust duct location from near the cabin ceiling to near the floor. Our results show a remarkable relationship between regions with a high aerosol encounter probability and airflow patterns in urban subway cabins, adding to our understanding of aerosol transmission. The proposed ventilation design showed a significant improvement in mitigating the infection risk associated with airborne transmission in a subway, in comparison with the commonly used ventilation designs, for all the source locations simulated in this study. To calculate the aerosol encounter probability inside the urban subway, we selected two surfaces that crossed the center of the passengers’ breathing zone hemisphere. For the case of the infected individual breathing near the fresh-air ducts, the conventional HVAC system design led to the largest spread and highest aerosol encounter probability (51.2% on the sitting sampling surface), but in the proposed design, this value was decreased to 3.5%. In the case of infected individuals breathing near the fresh-air ducts, the proposed design showed the most noticeable improvement compared to the conventional design. Based on our findings, we propose the following recommendations for risk mitigation in the subway considering that the ventilation design of the subway cannot be changed over a short period.

### 4.1. Concluding Remarks

1. Owing to the airflow directions, the safe zone is near the supply air ducts, and the hazardous zones are near the exhaust and recirculated ducts. Therefore, passengers should sit close to the air supply ducts and not stand inside the cabin. In other words, they should sit away from the exhaust and recirculate ducts.

2. With increased temperature and ACR of the supply air, the probabilities of aerosol encounter for all cases reduce.

3. Imperfect filtration of air in the HVAC system increase the probability for risk of aerosol encounter inside the urban subway.

### 4.2. Practical Recommendations

1. Based on the probabilities of encountering aerosols in the conventional HVAC system, authorities may provide alarm signs near an urban subway’s exhaust and recirculated ducts. To minimize face-to-face contact and reduce the risk of cross-infection, we propose providing separate doors for passengers entering or exiting the cabin. In some parts of the study, we assumed that the HEPA filter could capture all the viral particles. Regularly changing the HEPA filters is suggested to maintain their efficacy.

2. The authors encourage wearing a face mask to prevent the spread of respiratory droplets and aerosols within the urban subway and maintain social distancing while sitting.

The current study has several limitations. For the proposed HVAC system, we did not consider the dispersion of aerosols because of sneezing or coughing inside the cabin. Further research is required to better understand virus transmission via respiratory droplets in both HVAC systems. Researchers may focus on the head’s direction during coughing or sneezing inside the urban subway and study the high-value size of respiratory particles. An improved HVAC system model is required for future subway designs to manage viral transmission, keeping in mind the comfort of the passengers. Further research is needed to illustrate the energy consumption, thermal comfort, and aerodynamic behavior of urban subways. An improved HVAC system is necessary to mitigate aerosol transmission inside urban subways. To this end, this basic study must be experimentally validated.

## Figures and Tables

**Table 1:** The main characteristics of the evaluated case studies.

No. Case Study	Different Inputs	Common Inputs
The conventional HVAC system (Case studies 1, 2, 3, 4, 5 and 6)	The length and width of exhaust ducts are 2.5 m and 1.6 m, respectively. The number of exhaust ducts is 6,	Air change rate is 10 hr^−1^. The temperature values of the supply air, human breath, ambiance, and other cabin interior surfaces (such as mannequins, seats, and walls) are set at 20 °C, 30 °C, 25 °C, and 27 °C, respectively.
The proposed HVAC system (Case studies 1, 2, 3, 4, 5 and 6)	The length and width of exhaust ducts are 4 m and 0.6 m, respectively. The number of ducts is 12.	ν = 1.5 × 10^−5^ m^2^/s, T0 = 20 °C, β = 3 × 10^−3^ K^−1^, Pr = 0.71, Prt = 0.9, g = 9.81 m/s^2^, Sc = 1, Sct = 1, clothing insulation of mannequins is 0.60 clo.

**Table 2:** Comparison of the velocity and temperature with the results of the study by Tao et al. [56].

Values	Experimental Data	Present Work (RSM)	Present Work (*k–ε*)	Present Work (*k-ε* RNG)	Present Work (*k–ω*)	Present Work (*k–ω* SST)
*v_a_* (average velocity)	0.170	0.168	0.168	0.168	0.160	0.161
*v_x_* (horizontal velocity)	0.250	0.248	0.248	0.247	0.240	0.242
*v_z_* (vertical velocity)	0.590	0.578	0.578	0.578	0.562	0.560
*T_a_* (average temperature)	26.90	26.85	27.00	27.00	24.00	24.00
*T_x_* (horizontal temperature)	06.68	06.65	06.80	06.80	05.71	05.81
*T_z_* (vertical temperature)	06.94	06.94	07.02	07.02	06.07	06.20

## Data Availability

The data that support the findings of this study are available from the corresponding author upon reasonable request.
